# Hexavalent Molybdenum Reduction to Mo-Blue by a Sodium-Dodecyl-Sulfate-Degrading *Klebsiella oxytoca* Strain DRY14

**DOI:** 10.1155/2013/384541

**Published:** 2013-12-09

**Authors:** M. I. E. Halmi, S. W. Zuhainis, M. T. Yusof, N. A. Shaharuddin, W. Helmi, Y. Shukor, M. A. Syed, S. A. Ahmad

**Affiliations:** ^1^Department of Biochemistry, Faculty of Biotechnology and Biomolecular Sciences, Universiti Putra Malaysia, 43400 Serdang, Selangor, Malaysia; ^2^Department of Microbiology, Faculty of Biotechnology and Biomolecular Sciences, Universiti Putra Malaysia, 43400 Serdang, Selangor, Malaysia; ^3^Department of Bioprocess Technology, Faculty of Biotechnology and Biomolecular Sciences, Universiti Putra Malaysia, 43400 Serdang, Selangor, Malaysia

## Abstract

Bacteria with the ability to tolerate, remove, and/or degrade several xenobiotics simultaneously are urgently needed for remediation of polluted sites. A previously isolated bacterium with sodium dodecyl sulfate- (SDS-) degrading capacity was found to be able to reduce molybdenum to the nontoxic molybdenum blue. The optimal pH, carbon source, molybdate concentration, and temperature supporting molybdate reduction were pH 7.0, glucose at 1.5% (w/v), between 25 and 30 mM, and 25°C, respectively. The optimum phosphate concentration for molybdate reduction was 5 mM. The Mo-blue produced exhibits an absorption spectrum with a maximum peak at 865 nm and a shoulder at 700 nm. None of the respiratory inhibitors tested showed any inhibition to the molybdenum-reducing activity suggesting that the electron transport system of this bacterium is not the site of molybdenum reduction. Chromium, cadmium, silver, copper, mercury, and lead caused approximately 77, 65, 77, 89, 80, and 80% inhibition of the molybdenum-reducing activity, respectively. Ferrous and stannous ions markedly increased the activity of molybdenum-reducing activity in this bacterium. The maximum tolerable concentration of SDS as a cocontaminant was 3 g/L. The characteristics of this bacterium make it a suitable candidate for molybdenum bioremediation of sites cocontaminated with detergent pollutant.

## 1. Introduction

The role of bacteria in remediation of toxic compounds has been documented over the years and would continue to be a dominant technology for the remediation of organic and inorganic compounds [1–6]. The remediation of inorganic compounds such as heavy metals remains problematic due to the indestructible property of heavy metals. Microbes, however, utilize various mechanisms such as biosorption, bioprecipitation, efflux pumping, and bioreduction to counter the toxicity of metal ions. The microorganisms involved in the process come from a variety of genera. Metals that could be detoxified include molybdenum, mercury, lead, arsenic, uranium, copper, bismuth, selenium, chromium, and tungsten [7]. Amongst these metals, molybdate reduction by microbes has been reported one hundred years ago [7, 8].

However, detailed studies on the potential mechanism of reduction were initiated only in the past 25 years in *E. coli* [9], *T. ferrooxidans* (now *Acidithiobacillus ferrooxidans*) [10, 11], and the local bacteria *Enterobacter cloacae* strain 48 or EC 48 [12–15], *Serratia* spp. [6, 11, 16], *Enterobacter *sp. [17], *Acinetobacter calcoaceticus *[18], *Pseudomonas* sp. [19], and *Klebsiella* sp. [20]. The usage of bacteria in the bioremediation of molybdenum has been documented. In Tyrol, Austria, molybdenum pollution is caused by industrial effluents and has contaminated large pasture areas, reaching as high as 200 ppm causing scouring in ruminants [21]. Molybdate bioremediation using indigenous microbe from the contaminated site has shown positive results [21] and the works have opened the possibility of molybdenum bioremediation in other parts of the world.

The current documented reports show that metals' pollution in Malaysia is in the areas with heavy industrialization and scrap metal yards [6]. Aside from this, metal sludge, spent catalyst, spent lubricant, ink and the waste from the paint industries are also the major sources of molybdenum pollution [11]. Often cocontamination of organics and inorganics in wastes makes it difficult to remediate them. Hence, many workers have turned their attention to microbes with multiple biodegradation capacity. In this work, we report on the ability of an SDS-degrading bacterium [6] to reduce molybdenum to molybdenum blue. The characteristics of this bacterium make it a suitable candidate for molybdenum bioremediation of sites co-contaminated with detergent pollutant.

## 2. Materials and Methods

### 2.1. Isolation of Molybdenum-Reducing Bacterium

*Klebsiella oxytoca *strain DRY14 was originally isolated from an effluent of a car washing operator in Serdang, Selangor, Malaysia. The bacterium exhibits strong SDS-degrading capacity [6]. A bacterial colony grown on nutrient agar was streaked onto low phosphate (2.9 mM phosphate) agar media (pH 7.0) containing (w/v%) glucose (1.0), (NH_4_)_2_SO_4_ (0.3), MgSO_4_·7H_2_O (0.05), NaCl (0.5), yeast extract (0.05), Na_2_MoO_4_·2H_2_O (0.242), and Na_2_HPO_4_·2H_2_O (0.05). Glucose was autoclaved separately. In this study, molybdenum-reducing bacterium was isolated following the method described by Shukor et al. [22].

### 2.2. Assay for Molybdenum-Reducing Enzyme

Molybdenum-reducing enzyme was assayed using phosphomolybdate as the electron acceptor and NADH as the electron donor [22]. Briefly, laboratory-prepared ten to four phosphomolybdate or 10 : 4 ratios of phosphomolybdate were prepared arbitrarily as a 60 mM stock solution in deionized water. This was achieved by mixing molybdate (Na_2_MoO_4_·2H_2_O) and phosphate (Na_2_HPO_4_·2H_2_O) to the final concentrations of 600 and 240 mM, respectively. The pH of the solution was adjusted to pH 5.0 using 1 M HCl. This solution is stable at this pH for several months.

### 2.3. Crude Enzyme Preparation

High phosphate medium was chosen as the growth media for strain DRY14 since growth on low phosphate resulted in a blue sticky culture that complicated the preparation of crude enzyme and enzyme assay. This phenomenon also prevented crude enzyme preparation from EC 48 [12]. Although the high phosphate inhibits molybdate reduction to Mo-blue, the cells contain active enzymes. Bacteria were grown in one liter of high phosphate media at 30°C for 24 hours on an orbital shaker (100 rpm). The following experiment was carried out at 4°C unless stated otherwise. Cells were harvested through centrifugation at 10000 g for 10 minutes. Cells were washed at least once with distilled water, resuspended, and recentrifuged. The pellet was reconstituted with 10 mL of 50 mM Tris HCl buffer (pH 7.5) (Tris buffer prepared at 4°C) containing 0.5 mM dithiothreitol and 0.1 mM PMSF (phenylmethanesulfonyl fluoride) as an antiproteolytic agent. Cells were sonicated for 1 minute on an ice bath with 4-minute cooling until a total sonication time of at least 20 minutes was achieved. The sonicated fraction was centrifuged at 10,000 g for 20 minutes and the supernatant consisting of the crude enzyme fraction was taken.

### 2.4. The Effects of Respiratory Inhibitors and Metal Ions

Respiratory inhibitors such as Antimycin A, sodium azide, rotenone, and potassium cyanide were prepared as 20 mM, 50 mM, 50 mM, and 60 mM stock solutions, respectively, in deionised water. Antimycin A and rotenone were dissolved in acetone. Metal ions such as Fe^3+^ (FeCl_3_·6H_2_O, BDH), Fe^2+^ (Fe(NH_4_)_2_(SO_4_)_2_·6H_2_O, BDH), Cr^6+^ (K_2_Cr_2_O_7_, BDH), Mg^2+^ (MgCl_2_, BDH), Zn^2+^ (ZnCl_2_, BDH), Co^2+^ (CoCl_2_·6H_2_O, BDH), Cd^2+^ (CdCl_2_·H_2_O, SparkChem), Ag^+^ (AgNO_3_, JT Baker), Pb^2+^ (PbCl_2_, JT Baker), Mn^2+^ (MnCl_2_·4H_2_O, JT Baker), Sn^2+^ (SnCl_2_·2H_2_O, BDH), Cu^2+^ (CuSO_4_·5H_2_O, JT Baker), Ni^2+^ (NiCl_2_·6H_2_O, BDH), and Hg^2+^ (HgCl_2_, JT Baker), were dissolved in 20 mM Tris HCl buffer (pH 7.0) and added to the enzymatic reaction mixture at the final concentration of 2 mM. Inhibitors were added into the enzyme assay mixture to the final concentrations of 1.2, 10, 10, and 0.2 mM, respectively, in a volume not exceeding 20% of assay volume to prevent shifting in assay pH. Inhibitors and metal ions were preincubated with one hundred microlitres of enzyme in the reaction mixture at 4°C for 10 minutes minus NADH. The incubation mixture was then warmed to room temperature and NADH was added to start the reaction. Deionised water was added so that the total reaction mixture was 1.0 mL. As a control, 50 mL of acetone was added in the reaction mixture without inhibitors. The increase in absorbance at 865 nm was measured after a period of 5 minutes using a Shimadzu 1201 UV-Vis-NIR Spectrophotometer.

### 2.5. Experiment to Distinguish between Chemical and Enzymatic Reduction of Molybdenum

This experiment is a modification of the works carried out by Shukor et al. [14]. Briefly, bacteria were grown in 250 mL high phosphate media (100 mM) for 24 hours in several 250 mL conical flasks with shaking at 150 rpm on an orbital shaker (Yihder, Taiwan) at room temperature. Cells were harvested by centrifugation at 15,000 g for 10 minutes and the pellet resuspended in low phosphate solution (pH 7.0) containing (w/v) (NH_4_)_2_SO_4_ (0.3%), MgSO_4_·7H_2_O (0.05%), NaCl (0.5%), yeast extract (0.05%), and Na_2_HPO_4_ (0.05%). About 8 mL of this suspension was then placed in dialysis tubing previously boiled for ten minutes (1000 molecular weight cut-off) and immersed in sterile 100 mL of low phosphate media (pH 7.0) as described previously. Aliquots (1 mL) of the media were taken at the beginning of the experiment and after a static incubation period of 4 hours at room temperature and then read at 865 nm. At the same time, 1 mL was taken out from the content of the dialysis tubing and centrifuged at 15,000 g for 10 minutes. The supernatant was then read at 865 nm. Experiments were carried out in triplicate.

### 2.6. Statistical Analysis

All replicated data are represented as means ± SE (standard error). All data were analyzed using GraphPad Prism version 3.0 and GraphPad InStat version 3.05. Comparison between groups was performed using a Student's *t*-test or a one-way analysis of variance with post hoc analysis by Tukey's test. *P* < 0.05 was considered statistically significant.

## 3. Results 

### 3.1. Comparison of Mo-Blue Production among Molybdenum-Reducing Isolates

**Figure 1:**
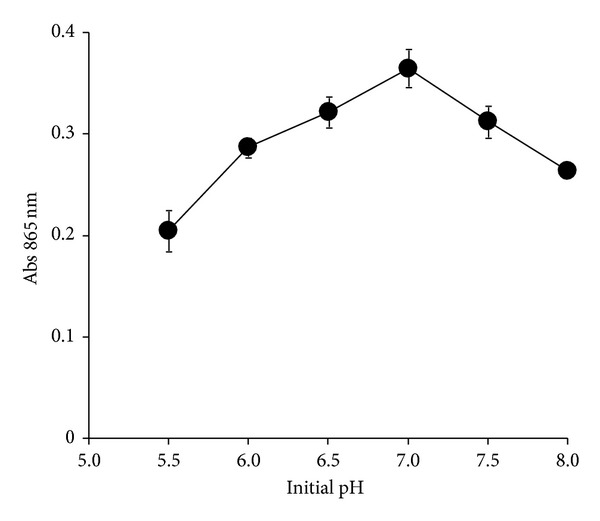
Molybdate reduction at various initial pH values. Strain DRY14 was grown for 24 hours in 50 mL of low phosphate liquid medium containing 10 mM molybdate at various initial pH values. Molybdate reduction was considered negligible if the absorbance at 865 nm is below 0.020. Error bars represent mean ± standard error (*n* = 3).

Strain DRY14 produced 1.4, 1.6, 1.9, 2.2, 2.2, 2.6, 5.3, 7.1, and 15.4 times more Mo-blue compared to *Serratia *sp. strain Dr.Y8, *S. marcescens *strainDr.Y9, *Serratia *sp. strain Dr.Y5, *Pseudomonas *sp. strain DRY2, *Enterobacter *sp. strain Dr.Y13, *Acinetobacter calcoaceticus *strain Dr.Y12*, Serratia marcescens* strain DRY6*, Enterobacter cloacae* strain 48,* and Escherichia coli* K12, respectively, with the exception of strain hkeem (Table 1). The optimum temperature supporting optimal molybdenum reduction was in between 25 and 30°C. The optimum initial pH for molybdate reduction was 7.0 (Figure 1).

### 3.2. The Effect of Electron Donor Sources

**Figure 2:**
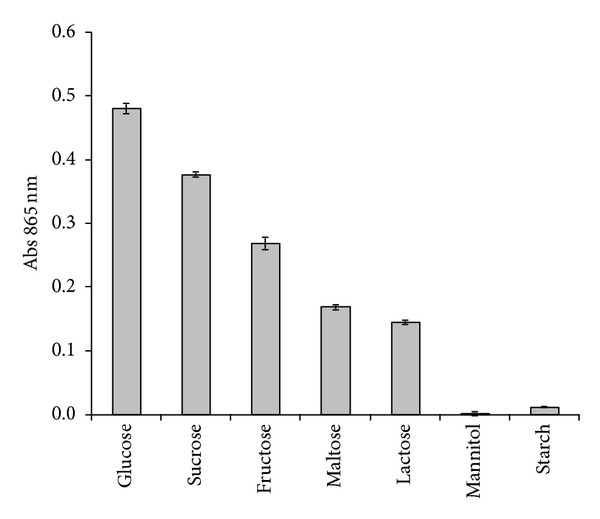
Molybdate reduction using various electron donors. Strain DRY14 was grown for 24 hours in 50 mL of low phosphate liquid medium containing 10 mM molybdate and various electron donors at the final concentration of 0.2% (w/v). The nitrogen source was 0.3% (w/v) ammonium sulphate. Molybdate reduction was considered negligible if the absorbance at 865 nm is below 0.020. Error bars represent mean ± standard error (*n* = 3).

Different electron donor sources such as glucose, sucrose, fructose, maltose, lactose, mannitol, and starch were used at an initial concentration of 0.2% (w/v) to study their effects on the molybdate reduction efficiency of the bacterium. Previous works have shown that Mo-blue production of bacteria requires simple assimilable carbon source as electron donors [11–20], and hence these carbon sources were used in this study. Of these, only glucose, sucrose, fructose, maltose, and lactose supported molybdate reduction after 24 hours of incubation with glucose supporting significantly more Mo-blue than the rest (*P* < 0.05) (Figure 2). Optimum concentration of glucose for supporting molybdate reduction was 1.5% (w/v) after 24 hours of static incubation. Further increase in glucose concentrations had a negative effect on molybdate reduction (data not shown). During cellular reduction of molybdate to Mo-blue, it was noted that cells precipitated together with a portion of the Mo-blue product preventing accurate cellular growth kinetic determination to be carried out in the presence of different nitrogen or electron donors.

### 3.3. The Effect of Phosphate and Molybdate Concentrations on Molybdate Reduction

**Figure 3:**
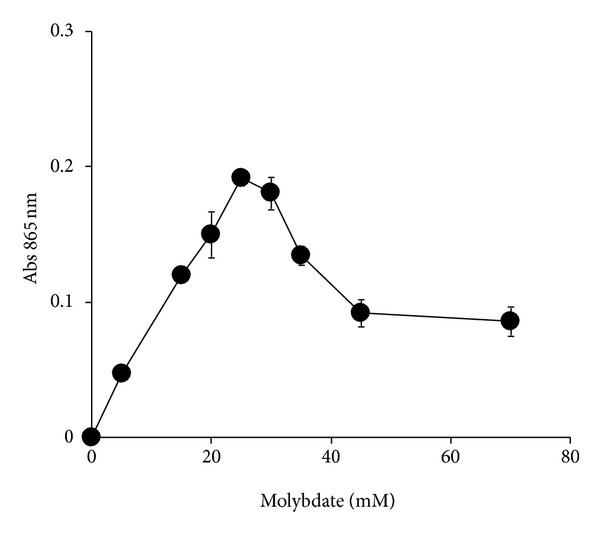
The effect of molybdate concentrations on molybdate reduction. Molybdate reduction was considered negligible if the absorbance at 865 nm is below 0.020. Error bars represent mean ± standard error (*n* = 3).

The study of the effect of molybdate concentrations on molybdate reduction was carried out in an increment of 5 mM. Molybdate reduction was found to increase linearly as molybdate concentration was increased from 0 to 25 mM and reached an optimum in between the concentrations of 25 and 30 mM. At higher concentrations, molybdate reduction was inhibited (Figure 3). The optimum phosphate concentration for molybdate reduction (set at 25 mM) in this bacterium was 5 mM (data not shown).

### 3.4. Mo-Blue Absorbance Spectrum

**Figure 4:**
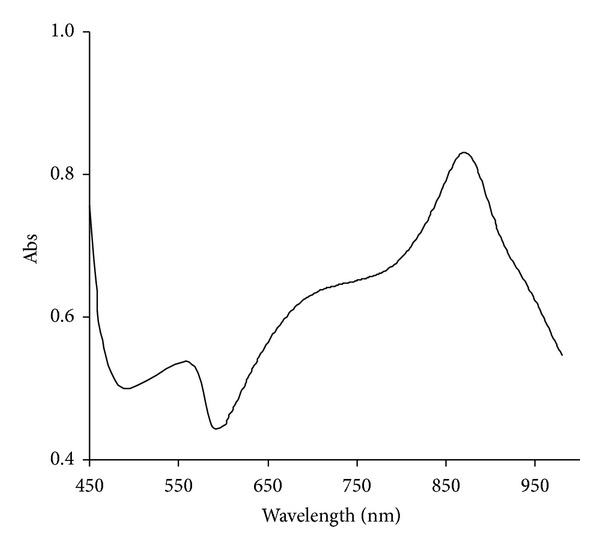
Scanning spectrum of Mo-blue after 24 hours of static incubation.

The Mo-blue produced by this isolate exhibited a unique characteristics absorption profile with a maximum peak at 865 nm and a shoulder at 700 nm. There was intense absorption near the ultraviolet region (Figure 4). We observed that during the progress of molybdate reduction, there was an increase in an overall absorption profile especially at the peak maximum at 865 nm and the shoulder at 700 nm in direct correlation with the increasing blue intensity of the media.

### 3.5. The Effect of Respiratory Inhibitors and Metal Ions on Molybdenum-Reducing Activity

In this work, none of the respiratory inhibitors tested show any inhibition of more than 10% to the Mo-reducing activity in this bacterium (data not shown). At first, it was contemplated that the dissimilar assay composition employed in the new assay system might influence the outcome of inhibitors' results. Thus, we make use of the original assay using molybdate as the electron acceptor substrate. We observed no inhibition on the molybdenum-reducing activity (data not shown). Heavy metals could generally inactivate enzymes, and hence, elevated presence of heavy metals could be a major problem in bioremediation. In this work, it was found that chromium, cadmium, silver, copper, mercury, and lead caused approximately 77, 65, 77, 89, 80, and 80% inhibition of the molybdenum-reducing activity, respectively, while other metal ions did not show inhibition (Table 2). Ferrous and stannous ions markedly increased the activity of molybdenum-reducing activity in this bacterium.

### 3.6. Experiment to Distinguish between Chemical and Enzymatic Reduction of Molybdenum

In order to provide further confirmation on the participation of enzyme in molybdate reduction in this bacterium, we enclosed this bacterium in dialysis tubing and immersed the tubing in low phosphate media and discovered more than 95% of the Mo-blue trapped in the dialysis tubing (data not shown).

### 3.7. Reduction of Mo-Blue in the Presence of SDS

**Figure 5:**
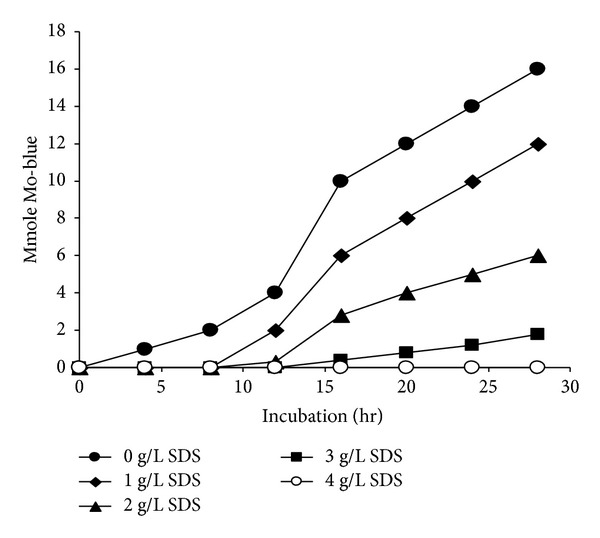
The effect of SDS on Mo-reduction after static incubation for 24 hours at molybdate concentration of 25 mM. Error bars represent mean ± standard error (*n* = 3).

Figure 5 shows the effect of SDS on molybdenum reduction. The carbon source was glucose at 1.5% (w/v). Reduction was not supported when SDS was used as the sole carbon source. Production of Mo-Blue was inhibited by SDS concentration greater than 1 g/L. Production was negligible at SDS concentration higher than 4 g/L.

## 4. Discussion

In all of the previous studies on molybdate reduction to molybdenum blue, it was observed that the reduced products cling so tightly to the bacterial biomass that it becomes impossible to determine cellular numbers and to carry out kinetic studies [6, 11, 12, 16–19, 23]. This observation was not reported in *E. coli* K12 [9]. The study of the effect of various parameters such as source of electron donors, temperature, molybdate, and phosphate on molybdate reduction is important. This knowledge not only is important for contributing to the fundamental understanding of the mechanism of reduction but also will be beneficial in the area of bioremediation of molybdenum especially with pH and source of electron donors as these parameters can be controlled by addition of suitable compounds into the soil.

Glucose appears to be the main carbon source optimal for supporting Mo-reduction [6, 9, 17–19]. In contrast, it was reported that sucrose is the best source of electron donor for EC 48, *S. marcescens *strain DrY6, and *Serratia* sp. strain Dr.Y5 [11, 12, 16]. Fructose was the best carbon source in strain hkeem [20]. Other carbon source that support Mo-reduction were glycerol, galactose, mannose, mannitol, maltose, lactose, raffinose, and sorbitol. Attempts to use the Biolog system as carbon sources for molybdate reduction were not successful in this work and other works [6, 17–19] since the colour produced from bacterial reduction of the formazan dye was more intense than that the Mo-blue produced. Our current works include optimizing this system to exploit the 95 carbon sources available in the system. It was previously demonstrated in EC 48 that molybdate reduction is growth-associated [24] and this is also true for several other Mo-reducing bacteria [11]. Hence, it is not surprising that glucose supported the highest reduction as it is the best substrate for growth and source of carbon as well as the best substrate for producing reducing equivalents in the form of NADH or NADPH. Both reducing equivalents are needed as substrates for chromate reductase [25] and the molybdenum-reducing enzyme [22]. However, in terms of cost effectiveness, sucrose in the form of cane and sugar molasses is preferred to pure and simple carbohydrates as it is a cheaper alternative found in abundance in industrial wastes in Malaysia [26]. Molasses is regularly used in works on microbiological chromate reduction [27]. Thus, sucrose in the form of molasses would be employed for future molybdenum bioremediation.

The study of temperature optimal for the growth of microbes would be very useful for bioremediation and maximizing enzyme yield for purification purposes [28]. The optimal temperature supporting molybdenum reduction in strain DRY14 differs markedly to all of the Mo-reducing bacteria studied so far. *Escherichia coli *strain K12 reduces molybdenum optimally between 30 and 36°C [9]. Ghani et al. reported 30°C as the optimum [12]. Other Mo-reducing bacteria isolated so far have optimal pH between 30 and 37°C. Although generally it is not possible to change temperature when performing bioremediation works on the field, screening for indigenous microbes for local bioremediation works is the norm since these microbes would have an optimum temperature close to the temperature of the site chosen for bioremediation. The optimal initial pH supporting reduction is also shared by the majority of the Mo-reducing bacteria [6, 9, 11, 12, 16–20]. The obligation for neutral pH and a moderate temperature range ensures that bioremediation treatments will be economical [29] and these observable facts are also shared by many chromate-reducing bacteria [25, 30, 31]. However, most soils with active metabolic activity usually exhibited lowering in pH due to several factors such as carbohydrate fermentation and carbon dioxide production leading to the lowering of pH [32].

Campbell et al. were the first to note the inhibitory effects of elevated concentrations of both phosphate and molybdate ions on bacterial molybdenum blue production [9]. Hence, it is very important to ascertain the effects of phosphate and molybdate ions on molybdate reduction in this bacterium. A similar ratio of phosphate to molybdate is seen in *E. coli* K12 and *Klebsiella* sp. strain hkeem [20], where, at 5 mM phosphate, 80 mM molybdate is the optimum concentration for supporting molybdate reduction [9]. In EC 48, the optimum ratio is 5 mM phosphate to 20 mM molybdate. In all of the Mo-reducing bacteria studied so far, phosphate concentrations higher than 5 mM inhibited molybdate reduction. Phosphate disrupts the phosphomolybdate complex preventing reduction to Mo-blue [33–36]. The highest reported concentration of molybdenum as a pollutant is at 2000 ppm (20.8 mM molybdate) from a molybdenum mine runoff [37]. At this concentration, all of the bacteria studied so far can reduce molybdate provided that the soil phosphate concentrations do not exceed 20 mM for appreciable reduction to take place. Fortunately, phosphate soil concentrations rarely exceeded this value in [38].

The increase at the peak maximum at 865 nm and more importantly the overall shape of the Mo-blue spectrum are the same as the majority of the Mo-reducing bacteria isolated so far [6, 11, 16–20, 23] indicating that a unique reduced phosphomolybdate species is involved in all of them. Incidentally, the spectrum is also similar to the Mo-blue spectrum produced by the ascorbic-acid-reduced phosphomolybdate in the phosphate determination method with a peak maximum at 880 nm and a shoulder around 700 nm. In a slight contrast, the Mo-blue spectrum from *E. coli* K12 showed a broad maximum peak at 820 nm and a shoulder between 600 and 700 nm [9] suggesting that a different species of phosphomolybdate probably had formed. The Mo-blue absorption spectrum is not similar to the absorption spectra of other Mo-blue products such as silicomolybdate and sulfomolybdate. This event is suggested to be an important event for phosphomolybdate formation [18, 19, 23]. Identification of phosphomolybdate by analysing the scanning spectroscopic profile is an accepted method but would not be enough to distinguish the many subtypes and lacunary species of phosphomolybdate. Identification of the exact phosphomolybdate species must be carried out using NMR and ESR. However, it would be generally enough to distinguish between phosphomolybdate and silicomolybdate or sulfomolybdate [39–42]. Based on the results above, works on the bacterial reduction of molybdenum to Mo-blue should take into account the pivotal role played by phosphomolybdate.

All of the inhibitors tested in this work show inhibition at the electron transport system at specific locations. The concentrations of inhibitors used in this assay are at least five times more than the suggested concentrations that would normally cause more than 50% inhibition of activity per mg of protein [43]. Rotenone is an inhibitor to NADH dehydrogenase while sodium azide and cyanide are inhibitors to the terminal cytochrome oxidase. Antimycin A inhibits cytochrome b [43]. The use of the respiratory inhibitor cyanide has been instrumental in locating the site of molybdenum-reducing activity in EC 48. The activity is suggested to be located at the electron transport system downstream from cytochrome b [12]. The results suggest that the electron transport system of this bacterium is not the site of molybdate reduction.

The rest of the metal ions did not have an effect on the molybdenum-reducing activity from this bacterium. Ferric ion did not enhance the activity of the molybdenum-reducing activity. Ferric, stannous, nickel, zinc, cobalt, ferrous, and silver enhanced the activity of the molybdenum-reducing enzyme in EC 48 severalfold while cupric and chromium ions strongly inhibited the activity of the enzyme [12]. Chromium also strongly inhibited Mo-blue production in *E. coli* K12 [9] while studies on the effects of metal ions on molybdate reduction were not carried out in *S. marcescens* strain DrY6 [11]. However, when we added each metal ion in the reaction mixture minus the crude extract of the enzyme, we discovered that molybdenum blue is produced by ferrous and stannous ions indicating that chemical reduction has occurred. The stimulatory effect of stannous and ferrous ions was similarly observed in this bacterium. We found out that this chemical reduction was only seen when the molybdate stock solution was adjusted to pH 7.0. When we measured the pH of the reaction mixture with the molybdate stock solution not adjusted to pH 7.0, we found the pH to be highly alkaline. Incidentally, ferrous and stannous ions have been used as reducing agents for the conversion of molybdate to Mo-blue in the phosphate determination method [33–36]. Even the stannous ions were used as a chemical reductant for the construction of the Mo-blue standard curve [12, 44]. This is likely the reason as to why ferrous irons were found to reduce molybdate to Mo-blue in the acidic media of *T. ferrooxidans *[10]. Hence, studies on the effect of metal ions on molybdate reduction and perhaps on other metal reduction works must be conducted with adequate control experiments.

We have shown recently that phosphate and arsenate are not physiological inhibitors towards Mo-blue production in EC 48 as both of these ions also inhibited the chemical production of Mo-blue. Only mercury was found to be a physiological inhibitor towards molybdate reduction in EC 48 [22]. The inhibition of molybdenum reduction to Mo-blue by copper and mercury could present a major problem for bioremediation of molybdenum polluted sites as these sites usually contain heavy metals at concentrations high enough to inhibit molybdate reduction. Both of these ions have also been reported to inhibit chromate reductase with the target of inhibition suggested as the thiol group [45]. The enormous amount of energy required for multitoxic metal resistance suggests that it would be difficult to isolate bacteria with such a capability. However, it would be important to screen and isolate bacteria with as many metal resistance capabilities as possible since the success of a bacterium to remediate a target metal would depend upon its resistance to other toxic nontarget metal ions present in the site [25].

The dialysis tubing experiment was carried out because molybdenum reduction to molybdenum blue can be carried out by many organic and inorganic reducing agents including ascorbic acid, dithionite, and metal ions. The reducing effect of stannous and ferrous ions seen in this work excellently emphasizes this point. This method is a modification of works carried out by Munch and Ottow originally to prove that the reduction of ferric to ferrous ions by bacteria is enzymatically mediated [46]. This method works because Mo-blue is colloidal and would not diffuse appreciably or slowly through dialysis tubings [35]. The dialysis tubing method was chosen since the conventional method of boiling cells (and cell fractions) to prove enzymatic reduction does not take into account that metal-reducing chemicals can be produced enzymatically. Boiling the cells denatures the enzymes responsible both for metal reduction and the enzymes that produce bioreductants that might be responsible for the reduction of metal ions. Thus, distinguishing between chemical and enzymatic reduction will not be possible. The results obtained reiterate the fact that molybdenum reduction, at least in the heterotrophic bacteria, is enzyme-driven and not due to biotic or abiotic reducing agents. The remaining 5%–10% of Mo-blue seen in the external side of the dialysis tubing is due to slow diffusion of the colloidal Mo-blue [6, 11, 16–19, 23].

Strain DRY14 can utilize SDS as the sole carbon source. However, reduction was not supported by SDS. Our results showed that reduction using glucose was inhibited by SDS. Previously this strain exhibited optimal growth on SDS at 2 g/L. As molybdenum reduction is growth-associated, the inhibitory effect of SDS on reduction is probably a result of growth retardation due to the toxicity of SDS.

In conclusion, the work carried out in this study is the continuation of the works reported more than one hundred years ago. The bacterium shows similar and differential Mo-reducing characteristics to previously reported Mo-reducing bacteria but with an added advantage—its ability to degrade SDS. More Mo-reducing bacteria should be reported to suit the various environmental conditions needed for remediation. Unfortunately, very few molybdenum-reducing microbes have been reported unlike works on chromate. Molybdenum is very toxic to ruminant and its remediation in contaminated aquatic bodies and soils of agricultural importance is highly sought. We are purifying the molybdenum-reducing enzyme from this bacterium to answer some of the questions pertaining to molybdate reduction to Mo-blue.

## Figures and Tables

**Table 1:** Amount of molybdenum blue produced from a 24-hour static culture of strain DRY14 in comparison with other Mo-reducing bacteria [20].

Bacteria	Micromole Mo-blue
*Klebsiella oxytoca *strain* DRY14 *	15.24 ± 0.21
*Klebsiella *sp. strain hkeem	32.81 ± 0.33
*Serratia *sp. strain Dr.Y8	10.53 ± 0.47
*S. marcescens* strain Dr.Y9	9.82 ± 0.24
*Serratia *sp. strain Dr.Y5	7.9 ± 0.12
*Pseudomonas *sp.strain DRY2	6.97 ± 0.78
*Enterobacter *sp.strain Dr.Y13	6.92 ± 0.24
*Acinetobacter calcoaceticus *strain Dr.Y12	5.80 ± 0.15
*Serratia marcescens* strain DRY6	2.88 ± 0.01
*Enterobacter cloacae* strain 48	2.15 ± 0.73
*Escherichia coli* K12	0.997 ± 0.06

**Table 2:** Effect of metal ions on molybdate reduction (mean ± standard error, *n* = 3).

Metal ions (2 mM)	Mo-blue produced (nmole/min/mg)
Control	45.01 ± 0.94
Cr^6+^	10.19 ± 0.76
Fe^3+^	45.50 ± 2.78
Fe^2+^	88.20 ± 2.29
Zn^2+^	45.58 ± 1.09
Mg^2+^	43.92 ± 1.26
Co^2+^	44.26 ± 1.16
Ni^2+^	41.56 ± 1.24
Cd^2+^	15.65 ± 0.04
Ag^+^	10.44 ± 0.10
Mn^2+^	43.09 ± 1.16
Cu^2+^	5.10 ± 0.24
Hg^2+^	9.20 ± 2.42
Pb^2+^	9.10 ± 0.25
Sn^2+^	94.95 ± 3.38
